# The Interplay of Renin-Angiotensin System and Toll-Like Receptor 4 in the Inflammation of Diabetic Nephropathy

**DOI:** 10.1155/2020/6193407

**Published:** 2020-04-30

**Authors:** Qi Feng, Dongwei Liu, Yanfang Lu, Zhangsuo Liu

**Affiliations:** ^1^Department of Nephrology, The First Affiliated Hospital of Zhengzhou University, Zhengzhou 450052, China; ^2^Research Institute of Nephrology, Zhengzhou University, Zhengzhou 450052, China; ^3^Key Laboratory of Precision Diagnosis and Treatment for Chronic Kidney Disease in Henan Province, Zhengzhou 450052, China; ^4^Core Unit of National Clinical Medical Research Center of Kidney Disease, Zhengzhou 450052, China

## Abstract

Diabetic nephropathy (DN) is one of the most serious chronic kidney diseases and the major cause of end-stage renal failure worldwide. The underlying mechanisms of DN are complex and required to be further investigated. Both innate immunity and renin-angiotensin system (RAS) play critical roles in the pathogenesis of DN. Except for traditional functions, abnormally regulated RAS has been proved to be involved in the inflammatory process of DN. Toll-like receptor 4 (TLR4) is the most deeply studied pattern recognition receptor in the innate immune system, and its activation has been reported to mediate the development of DN. In this review, we aim at discussing how dysregulated RAS affects TLR4 activation in the kidney that contributes to the exploration of the pathogenesis of DN. Understanding the interplay of RAS and TLR4 in inducing the progression of DN may provide new insights to develop effective treatments.

## 1. Diabetic Nephropathy and Its Major Pathogenesis

Diabetic nephropathy (DN), one of the most important microvascular complications of diabetic mellitus (DM), is considered as the predominant cause of end-stage renal disease (ESRD) worldwide [1]. In 2019, the U.S. Renal Data System depicted that the number of diabetics improves quickly, which results in the rapid progression of DN and ESRD around the world [2]. A recent cross-sectional survey showed that the prevalence of DN in Chinese rural population was about 35.5%, and there was still an upward trend [3]. Although studies on the pathogenesis of DN have been steadily grown, the exact mechanism has not yet been well clarified. The basic pathological changes of DN are glomerulosclerosis, release of inflammatory mediators, and accumulation of extracellular matrix. The pathological manifestations of early DN are glomerular hypertrophy, glomerular and tubular basement membrane thickening, and progressive accumulation of mesangial extracellular matrix (ECM), and its later stage is glomerular and tubulointerstitial fibrosis, which eventually leads to proteinuria and renal failure [4].

Current studies generally believe that metabolic disorders, inflammation, oxidative stress, hemodynamic changes, and other factors are involved in the initiation and development of DN [5]. Renin-angiotensin system (RAS) is an important endocrine cascade to modulate and balance the blood pressure, volume homeostasis, water, and electrolyte and maintain the relative stability of the human internal environment. RAS activation is one major cause of renal injury in DN and is also suggested to drive a range of inflammatory processes in the kidney [6]. Clinical application of angiotensin-converting enzyme inhibitors (ACEIs) and angiotensin II receptor blockers (ARBs) to block the RAS system [7], as well as strict controlling of blood sugar and pressure, is a major method to treat and delay the development of DN. Although the above-mentioned methods can play a certain therapeutic effect, it still could not completely prevent the progression of DN to ESRD, indicating that it may contain other important pathogenesis except for the above mechanisms.

Since Hasegawa et al. firstly proposed in 1991 that inflammatory factors may be involved in the occurrence and development of DN [8], growing evidences have acknowledged that inflammation plays a key role in the progression of DN [9, 10]. Emerging studies found that RAS activation was involved in key events of the inflammatory process [11]. To some extent, DN is also accepted as a low-grade inflammatory disease. A large amount of clinical and experimental studies have shown an innate immune system, via its actions on Toll-like receptors (TLRs), associated with the pathophysiological process of DN [12]. Among all TLRs, TLR4 is the most extensively studied [13]. Numerous studies have reported the important role of TLR4 in stimulating the proinflammatory signaling cascade as well as participating in the pathogenesis of diabetic complications [14]. Thus, exploring the relationship of TLR4 and diabetic kidney injury has become a hot topic in the research field of DN. However, few studies focus on the possible correlation between dysregulated RAS components and activated TLR signaling pathway in mediating the inflammatory reactions in kidney [15]. In addition, the precise mechanism of dysregulated RAS components acting as an inhibitor or inducer for TLR activation in triggering the generation and progression of DN is still unclear. Therefore, in this review, we aim at providing a brief description about the interplay of activated TLR4 and RAS dysregulation, as well as speculating their possible interactive mechanisms in mediating inflammatory process of DN.

### 1.1. Innate Immune and Toll-Like Receptors (TLRs)

**Figure 1:**
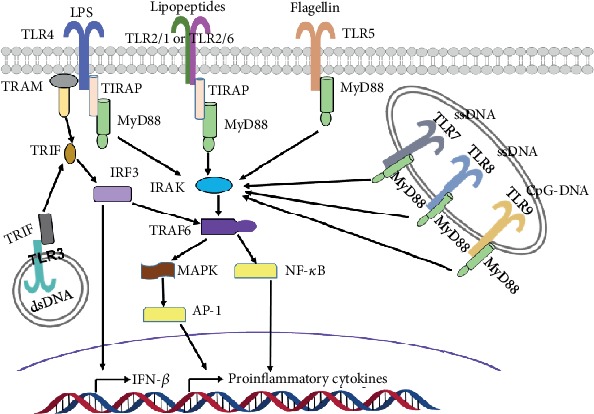
TLRs and signaling molecules on the basis of literatures. The majority of TLRs seem to function as a homodimer, but TLR2 forms a heterodimer with TLR1 or TLR6. TLR signaling consists of two distinct pathways: the MyD88-dependent and MyD88-independent cascades. TLR4 can activate both MyD88-dependent and MyD88-independent pathways. TLR3 in the endosome recruits TRIF and mediates the MyD88-independent pathway. The engagement of TLR7, TLR8, and TLR9 in the endosome membrane leads to the formation of a complex that consists of MyD88 and IRAK. TLR2, TLR7, TLR8, and TLR9 can only activate MyD88-dependent pathway. LPS, lipopolysaccharides; dsRNA, double-stranded RNA; ssRNA, single-stranded RNA; TIRAP, TIR domain-containing adaptor protein/MyD88 adaptor-like protein; TIR, Toll-like/IL-1 receptor; TRAM, TRIF-related adaptor molecule; TRIF, TIR-domain-containing adaptor-inducing IFN-*β*; IRF3, interferon regulatory factor 3; IRAK, interleukin-1 receptor-associated kinase; TRAF6, tumor necrosis factor receptor- (TNFR-) associated factor 6; MAPK, mitogen-activated protein kinase; AP-1, activator protein 1; NF-*κ*B, nuclear factor-*κ*B; IFN-*β*, interferon-*β*.

Innate immune system is the first line of defense against pathogenic microorganisms, which stimulates the innate immune response through pattern recognition receptors (PRRs). At present, several PRRs have been determined, including Toll-like receptors (TLRs) and nucleotide-binding oligomerization domain- (NOD-) like receptors (NLRs) [16, 17]. Among which, TLRs are the most widely studied. Up to now, 11 kinds of human TLRs (TLR1-TLR11) and 13 kinds of mice TLRs (TLR1-TLR13) have been found [18]. Except for presenting on different immune cells such as macrophages, lymphocytes, dendritic cells, and neutrophils, TLRs are also expressed in various organs including the heart, lungs, liver, and kidney, where they contribute significantly to various pathologies [19–21]. TLRs present on cell surface recognize extracellular pathogen-associated molecular patterns such as lipopolysaccharide, lipopeptides, and bacterial DNA, whereas intracellular TLRs identify endogenous danger-associated molecular patterns including viral or microbial nucleic acids and nonmicrobial origin [22]. TLR activation are initiated by the recognition of corresponding ligands, then the associated adaptor molecules are recruited, and the down-stream signaling cascades are evoked via the myeloid differentiation factor 88- (MyD88-) dependent and/or MyD88-independent pathways, eventually resulting in the activation of nuclear factor kappa-B (NF-*κ*B) and transcription factors as well as the production of proinflammatory cytokines and chemokines (Figure 1).

### 1.2. TLR4 and Inflammation in DN

Among all TLRs in human, TLR4 is the earliest discovered and the most extensively studied. TLR4 is widely expressed in intrinsic renal cells such as mesangial cells, tubular epithelial cells, and podocytes. Previous studies have shown that TLR4 not only does upregulate noninflammatory kidney disease such as renal ischemia-reperfusion injury [23], tubulointerstitial nephritis [24], and glomerulonephritis [25] but also is actively involved in the occurrence and progression of DN [26].

Increasing evidences propose that TLR4 activation is closely involved in the inflammatory process and renal fibrosis of DN [27]. Endogenous ligands play a crucial role in the initiation of the TLR-mediated immune response. Upon activation by binding the released endogenous ligands in immune and kidney cells, TLR4 initiates down-stream signaling cascades via MyD88-dependent and MyD88-independent pathways, which eventually leads to the activation of NF-*κ*B [28]. Activated NF-*κ*B subsequently transfers to the nucleus and induces the transcription and translation of related inflammatory mediator genes, resulting in increased release of proinflammatory cytokines and chemokines such as monocyte chemoattractant protein- (MCP-) 1, IL-6, IL-8, IL-18, tumor necrosis factor *α* (TNF-*α*) [29, 30]. As a biomarker of DN, NF-*κ*B is found to be involved in other pathologic processes of DN, including the advanced glycation end-product accumulation, activation of RAS pathways and protein kinase C (PKC), and oxidative stress [31–34].

**Figure 2:**
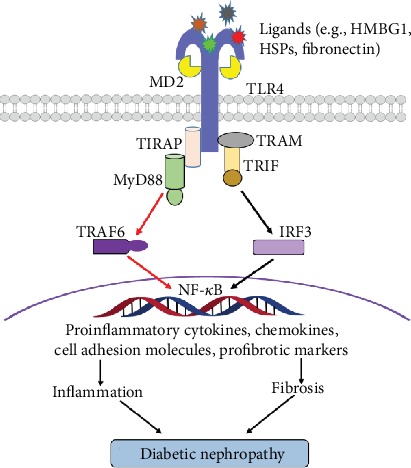
TLR4 signaling pathway in the DN on the basis of literatures. Activation of TLR4 initiates the MyD88-dependent and MyD88-independent pathways. Moreover, the pathways converge in the activation of NF-*κ*B, which is responsible for the synthesis and secretion of proinflammatory cytokines, chemokines, cell adhesion molecules, and profibrotic markers involved in inflammation and fibrosis, eventually leading to diabetic nephropathy. HSPs: heat shock proteins; HMGB1: high-mobility-group box 1.

Endogenous ligands are significantly upregulated in the presence of high glucose, hypoxia, and hyperlipidemia, which are central to the pathophysiology of DN. Growing evidence have emphasized that TLR4 is activated by the endogenous ligands and upregulated in the pathogenesis of DN (Figure 2). High-mobility group box 1 (HMGB1) is an endogenous ligand of TLR4. Lin et al. found an increase of HMGB1 in the proximal tubular cells of patients with DN [26]. Kim et al. revealed an upregulation of HMGB1 expression in glomerular and tubular epithelial cells in a DN mouse model along with increased NF-*κ*B activity [35]. Mudaliar et al. observed that high glucose increased the release of HMGB1, expression of TLR4, activation of NF-*κ*B, and secretion of cytokines. Furthermore, Dasu et al. demonstrated that the expression of TLR4 was significantly decreased by the inhibition of PKC activity and nicotinamide adenine dinucleotide phosphate (NADPH) oxidase. Under high-glucose conditions, TLR4^−/−^ monocytes displayed a remarkable decrease in NF-*κ*B activation [36]. Mudaliar et al. found that high-glucose stimulation increased the expression of TLR4 and enhanced the release of HMGB1 [37]. Mudaliar et al. also reported that high-glucose stimulation significantly upregulated TLR4 expression with increased NF-*κ*B activation, HMGB1, IL-8, and ICAM-1 production. Blockade of TLR4 could inhibit the NF-*κ*B activation and synthesis of proinflammatory cytokines and chemokines caused by HMGB1 induction [38]. Lin et al. displayed that blocking TLR4 with an inhibitor CRX-526 would exert renoprotective effects in eNOS knockout mice with DN [39]. Kuwabara et al. reported that hyperlipidaemia induced the up-regulation of S100A8 and the corresponding receptor TLR4 in the glomeruli of mice accompanied with DN [40]. Kaur et al. found that the activity and the expression level of TLR4 were enhanced under hyperglycemia in mouse mesangial cells that are related with DN [41]. Cha et al. demonstrated that inhibition of TLR4 signaling pathway could provide a direct protective function in mice with DN [42]. Jialal et al. proved that the global deficiency of TLR4 enabled to downregulate the renal inflammation, fibrosis, and podocytopathy that contribute in DN [43]. Taken these studies together, it thus indicates that the endogenous ligands enhanced an up-regulation in expression and signal transduction of TLR4 under high-glucose stimulation and exacerbate the development of DN in diabetic kidneys among human and animal models [44]. Additionally, knockout of TLR4 indicates a protection from characteristic features of DN in kidney. In fact, during the past decade, the number of endogenous ligands implicated in the pathogenesis of DN is ever growing. Numerous endogenous ligands have been identified for the activation of TLR4 in DN, and some of them have been shown to be closely associated with inflammation and renal fibrosis [14, 45]. Although the precise molecular mechanisms and contribution of TLRs in the pathogenesis of DN are still not well depicted, it strongly shows that endogenous ligands significantly contribute to the inflammation in DN via TLR4 signaling pathway.

### 1.3. Renin-Angiotensin System (RAS)

**Figure 3:**
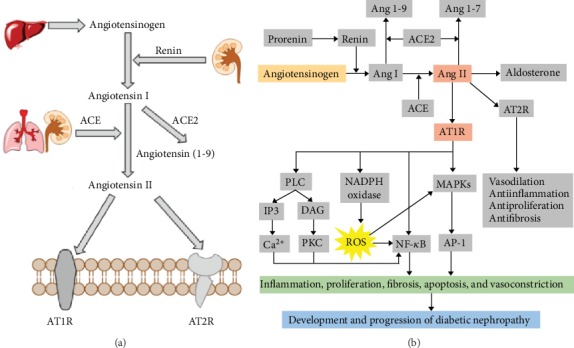
Classical renin-angiotensin system (RAS) and the overview of RAS under pathological conditions of DN. (a) Components of classical RAS. (b) Schematic representation of RAS signaling cascades involve in the inflammation of DN. Ang: angiotensin; ACE: angiotensin-converting enzyme; AT1R: angiotensin II type 1 receptor; AT2R: angiotensin II type 2 receptor; PLC: phospholipase C; IP3, inositol triphosphate; DAG, diacylglycerol; PKC, protein kinase C; MAPKs: mitogen-activated protein kinases; NADPH: nicotinamide adenine dinucleotide phosphate; ROS: reactive oxygen species.

Since renin was firstly identified in 1898, RAS has been extensively studied [46]. RAS is traditionally considered as one important humoral regulation system in our body and contains a series of peptide hormones and corresponding enzymes [47]. Classical RAS is comprised of angiotensinogen (AGT), renin, angiotensin I (Ang I), angiotensin I-converting enzyme-2 (ACE2), angiotensin I-converting enzyme (ACE), angiotensin II (Ang II), aldosterone, Ang II type 1 receptor (AT1R), and the Ang II type 2 receptor (AT2R) (Figure 3(a)). The classical RAS cascade starts with the synthesis of renin that originally from prorenin cleavage in the juxtaglomerular cells. AGT is the main precursor peptide of RAS, which can be subsequently cleaved by renin and ACE to form Ang I and Ang II. Compared to ACE, ACE2 removes one amino acid less from Ang I, thus resulting in the formation of angiotensin 1-9. Ang II is the main effector peptide of RAS system and exerts most of its physiological effects via the activation of AT1R and AT2R [48]. As the physiologically active component of RAS, Ang II binds to specific receptors in adrenal cortex, resulting in aldosterone release, which helps in sodium reabsorption in the kidney.

Systemic RAS realizes long-term blood pressure regulation by directly constricting blood vessels or slowly releasing aldosterone to expand the intravascular volume. During the past two decades, in addition to the systemic RAS, emerging evidences have recognized there is another RAS, called the local RAS. The local RAS has been identified to be distributed in a variety of organs and tissues like the heart, liver, kidney, brain, skeletal muscles, pancreas, retina, adipose, neuronal, and reproductive tissue, which might affect the physiological and pathological regulation of tissues, associated with or without the systemic RAS [49]. Up to now, accumulating evidences suggest that the activation of RAS plays an important role in regulating vasoconstriction, fluid balance, and blood pressure, as well as increasing oxidative stress, renal fibrosis, cell proliferation, and inflammatory process [50].

### 1.4. RAS Components Involve in the Inflammatory Process of DN

Clinical and experimental results have well documented that abnormal regulation of RAS is a major factor in the development of chronic kidney disease including DN. Blockade of RAS using either ACEIs or ARBs can alleviate disease progression in patients with DN [51]. However, this is only partially explained by their hemodynamic effects. Studies have revealed that RAS activation also involves in inflammatory process of chronic kidney disease via blood pressure-independent manner [52]. RAS activation in kidney with DN could affect inflammatory progression that results in activation and proliferation of different renal cells such as mesangial cells, endothelial cells, and podocytes, as well as immune cells including macrophages and lymphocytes [53].

The activation in RAS involves multiple components in the cascade and leads to deleterious effects on the development of DN (Figure 3(b)), including kidney injury and renal fibrosis [48]. Renin is expressed and secreted by juxtaglomerular apparatus (JGA) cells, and its expression and activity can be regulated by inflammatory signaling. For example, cytokines like IL-6, IL-1*β*, and TNF-*α* attenuated renin expression via MAPK/STAT/NF-*κ*B signaling pathways, respectively [54–56]. A further study indicated that high-glucose stimulation could increase the renin expression and renin might have Ang II-independent actions to develop renal fibrosis [57]. Therefore, people speculated that renin may be involved in modulating the expression of proinflammatory cytokines in chronic kidney disease including DN, but this hypothesis had not been established. ACE is generally served as a primary enzyme that catalyzes the synthesis of Ang II from Ang I. Only few studies revealed that ACE enabled to accelerate the inflammatory responses in the kidney. Anderson et al. found the total expression of ACE was significantly decreased in DM rats, with specific redistribution in kidney tissues with DN [58]. Ustundag et al. demonstrated that ACE activity levels were significantly higher in patients with DN than in type II DM patients without complication, which supported the hypothesis that ACE played an essential role in diabetic complications including DN [59]. Therefore, ACE blockade has been widely used in the clinic to slow the progress of DN. Ang II is the main effector peptide of RAS and originally served as a modulator in regulating circulatory homeostasis [60]. Recent studies have shown that Ang II, mainly via AT1R and AT2R, participates in the inflammation and fibrosis in the kidney. Ang II-mediated inflammatory process could directly induce the activation of NF-*κ*B in different intrinsic renal cells and local immune cells via different signaling pathways. Additionally, Ang II also directly stimulates the activation of PKC, protein tyrosine kinases (PTKs), mitogen-activating protein kinases (MAPKs), extracellular signal-regulated kinase (ERK), c-Jun amino terminal kinase (JNK), p38 MAPK, and the activator protein-1 (AP-1). All of these above molecules are proved to be involved in proliferation, differentiation, fibrosis, and inflammatory processes [61]. In addition to this direct action, chronically elevated Ang II could promote the generation of vascular oxidative stress, synthesis of reactive oxygen species (ROS), and activation of NADPH oxidase, which further induced the activation of NF-*κ*B and secretion of proinflammatory cytokines, chemokines, and fibrogenic factors, thus resulting in the occurrence of inflammation and fibrosis as well as acceleration of the development of DN [48]. Numerous studies have suggested Ang II activation contributes to the inflammatory responses and tissue remodeling of DN and thereby to the progression of kidney injury in DN [11]. Wolf et al. demonstrated that the effect of high-glucose stimulation was similar with angiotensin II treatment on the cell proliferation and cytokines release in cultured proximal tubular and glomerular mesangial cells [62]. Leehey et al. suggested that the high-glucose milieu of diabetes increased the Ang II production in the kidney, especially in renal mesangial cells, leading to the increase of TGF-*β* secretion and matrix accumulation. And it is thus supposed as an important mechanism linking hyperglycemia and Ang II in the pathogenesis of DN [63]. Lodha et al. revealed that under high-glucose stimulation, Ang II induced the development of renal injury in glomerulus mesangial cells by promoting cell apoptosis, inflammation, and extracellular matrix accumulation [64]. Therefore, previous data supports the hypothesis that Ang II is a key mediator of inflammation in the development of DN.

Ang II affects the renal damage and involves in the aldosterone secretion, fibrosis, inflammation, and oxidative stress of chronic kidney disease via its dominant receptor AT1R. However, whether Ang II activates the immune responses by directly stimulating AT1R remains inconsistent. Ogawa et al. suggested that increased oxidative stress was a powerful factor in promoting renal injury in DN and AT1R-mediated actions of Ang II played a central role in this process [65]. In clinical studies, ARBs could selectively block the AT1R and have been well documented to decrease albuminuria and improve cardiovascular remodeling, thereby reducing the progression of DN to ESRD.

As reviewed in this part, increased evidences have demonstrated that RAS activation seems to be associated with the inflammatory and vascular alterations. Furthermore, multiple components of the RAS signaling cascade are reported to influence the inflammation-induced kidney injury and renal fibrosis in the development of DN. A great deal of evidences have unraveled roles of RAS, and particularly, its main effector molecule Ang II participates in the inflammatory process of DN. Herein, we focus on RAS-mediated inflammatory responses involving the innate immune system. More recent studies demonstrate the upregulation of TLR4 in the pathogenic process of DN and several evidences suggest TLR4-dependent signaling pathway affects the proinflammatory effects of Ang II. Thus, in this review, we aim at discussing the contribution of the innate immune system via the interactions of TLR4 and the major RAS components within the kidney during DN.

### 1.5. The Interplay of TLR4 and RAS in the Inflammatory Process of DN

In the past few years, emerging evidences have however documented the new roles of RAS as proinflammatory molecule that contributes to the progressive deterioration of organ functions in kidney disease. More recent studies found that renal RAS activation was one major pathogenic factor in DN. Previous studies have proved the elevation of RAS components during DN and the beneficial effects of RAS blockade [66, 67]. Renal RAS contains all components of classical RAS, among which, Ang II, AT1R, and ACE are the most widely studied in chronic kidney disease. Current treatments of DN mainly focus on anti-RAS therapy by blocking Ang II, ACE, and AT1R, but only few studies have been performed to understand the local regulation of each RAS component by high-glucose stimulation, and the potential relations with inflammation are still unclear.

During DN, renal RAS is activated along with elevated production of local Ang II, AT1R, and ACE, which are elevated in tubular, interstitial, and fibroblast-like cells in the kidney. As a vasoactive peptide in RAS system, Ang II exerts a variety of physiological regulations, including controlling the renal blood flow and increasing the glomerular filtration rate [68]. Recently, Ang II is proved to be upregulated along with the activation of RAS in DN. Furthermore, Ang II is functional as a promoter to activate interstitial and tubular fibrosis as well as to trigger inflammation by elevating the production of inflammatory cytokines and adhesion molecules [69]. Ang II activation is also demonstrated to promote apoptosis, inflammation, and extracellular matrix accumulation, which results in mesangial cell damage [64]. Previous studies have well documented that Ang II-induced ROS contributes to renal inflammation. Activated Ang II, via binding to AT1R, enables to increase oxidative stress and promote the ROS production in DN by inducing the activation of NADPH oxidases to produce superoxide and hydrogen peroxide. Subsequently, ROS-sensitive signaling cascade is initiated, and it promotes the production of inflammatory cytokines and chemokines like TNF-*α*, IL-1*β*, IL-6, IL-12, and IFN-*β*, resulting in the renal injury [70]. Moreover, Ang II can also directly activate the transcription factor NF-*κ*B that is critical for initiating the coordinated expression of classical components during the DN inflammatory response [71]. Though accumulating evidences have shown that overexpressed RAS components induce the activation of NF-*κ*B and release of proinflammatory cytokines, the precise mechanism of RAS components, particular the Ang II-mediated inflammation in DN, is still not yet well elucidated.

DN is currently considered as a chronic kidney disease with low-grade inflammation. TLR4, an innate immune receptor, is considered to play an inevitable role in pathogenesis of DN. Several studies have found that the upregulation of TLR4 in many renal cells such as podocytes and tubular and interstitial cells. Verzola et al. proved that the activation of TLR4 played a critical role in the progression of DN [72]. Ma et al. found that TLR4 activation promoted podocyte injury and interstitial fibrosis that are associated with DN [73]. During the inflammatory process of DN, TLR4 activation could induce the production of pro-inflammatory cytokines and activation of NF-*κ*B via MAPK signaling pathway. And this is similar to the inflammatory reactions caused by RAS activation under high-glucose stimulation.

Except for the direct proinflammatory properties of RAS during pathogenic process, an indirect modulatory effect of RAS components on TLR4 has been proposed. A variety of evidences have demonstrated that Ang II exerts proinflammatory effects partly via increasing TLR4 expression by stimulating TLR4-mediated signaling pathway in various organs and cell types. Dange et al. found that brain TLR4 was involved in Ang II-induced hypertensive effects possibly acting as a protective molecule in the pathogenesis of Ang II-induced cardiovascular diseases [74]. Biancardi et al. reported a cross talk between AT1R and TLR4 in mediating Ang II-dependent microglial activation and oxidative stress within the PVN [75]. Wu et al. investigated that TLR4 expression was modulated by local Ang II in rat peritoneal mesothelial cells and caused the activation of NF-*κ*B signaling and induction of CD40, TNF-*α*, and IL-6 expression, which suggested that RAS, Ang II-forming pathway, may have potential in regulating the TLR4 expression [76]. Cheng et al. observed that AT1R inhibitor blocked the intimal neovessel density partly via reducing TLR4-mediated inflammatory responses and MMP activation [77]. Cai et al. indicated that Ang II-induced liver fibrosis could be inhibited by NLRP3 depletion, and Ang II upregulated the expression of NLRP3 inflammasome by activating the TLR4/MyD88/NF-*κ*B pathway [78]. Low-density lipoprotein (LDL) is a marker of endothelial dysfunction and the metabolic syndrome. Catar et al. found that LDL could upregulate the expression of RAS and TLR4 in a time-dependent manner in human endothelial cells, which suggested a putative link between TLR4, local RAS, and atherosclerosis caused by systemic lipoprotein [79]. All of the above descriptions have well documented the functional studies of RAS cross-linking with TLR4 in heart, brain, and liver diseases. However, the research about the interactions between RAS and TLR4 in stimulating the development of DN has just become a little apparent only recently. Furthermore, the associated reports have not been summarized.

Accumulating evidences have shown that Ang II exerts proinflammatory effects in the kidney. Moreover, under the stimulation of high glucose, RAS is activated along with the expression of upregulated TLR4. Therefore, Ang II is supposed as a “danger” factor by modifying TLR4 expression in mesangial cells. However, the specific molecular mechanism still needs to be further studied.

Ang II is considered as a potent mediator for renal inflammation and served as a potent inducer of ROS in renal injury. By binding to AT1R, Ang II activates the NADPH oxidase complex to produce superoxide and hydrogen peroxide as well as to increase the expression of ROS resulting in exerting proinflammatory properties in DN by activating NF-*κ*B signal pathway and inducing the expression of chemokines. In addition, ROS can also be modulated by TLR4-MyD88 signaling pathway; it is logical to speculate that Ang II may contribute to renal cell injury through TLR4-MyD88-mediated innate immune pathway. Candesartan is a highly selective inhibitor for AT1R, and it could inhibit the expression of chemokine, NF-*κ*B, and ROS as well as suppress the TLR4 expression in renal tubular epithelial cells. Therefore, it may indicate that candesartan attenuated Ang II-induced TLR4 signaling pathway to inhibit the ROS generation and apoptosis in the kidney [80, 81]. Lv et al. remarkably revealed that the TLR4-MyD88 signaling pathway made contributes to ROS generation and it might be involved in the process of Ang II-induced impairment in mesangial cells [82]. Wolf et al. described that Ang II could induce the innate immune responses by upregulating the TLR4 mRNA and protein in mouse mesangial cells, and this effect was mediated through AT1R [83]. Taken together, it showed that Ang II-upregulated TLR4 expression and signaling pathway might be dependent on AT1R activation. Subsequently, Bondeva et al. found that Ang II stimulated TLR4 expression both in mouse mesangial cells and podocytes, which contributed to renal inflammation [84]. In addition, Lv et al. reported a synergistic effects between Ang II and TLR4 in triggering inflammatory injury of rat mesangial cells under high-glucose conditions, which suggested that RAS activation might be another mechanism in mediating renal TLR4 activation in DN [85]. Pearse et al. demonstrated that ETS-1 was a critical transcription factor involved in the growth-promoting effects of Ang II. And Ang II increased ETS-1 expression in mesangial cells and glomerulus, which required NADPH oxidase-derived ROS, COX-2, and MAPKs. Ni et al. suggested that ETS-1 functional as a critical down-stream transcriptional mediator of Ang II-induced ROS generation by regulating the expression of NADPH oxidase. Roger et al. reported that transcription of the mouse TLR4 gene was found to be under the control of regulatory elements, including ETS-1. Taken together, it is speculated that Ang II-enhanced TLR4 signaling could regulate the expression of NADPH oxidases, and this process was through the transcription factors such as ETS-1 [86–88]. Lodha et al. revealed that ROS played a key role in Ang II-induced mesangial cell apoptosis, and reductive drugs could change this reaction. Matsuzawa et al. confirmed that TLR4 mediated ROS acted as transducers of apoptosis. Erkan et al. discovered there was a correlation between ROS production and the activation of apoptosis in proximal tubule cells. Together with these observations, it is suggested that Ang II-induced cell apoptosis might be due to the activation of TLR4 signaling pathway.

Except for the investigations about the Ang II may closely regulate the TLR4 signaling pathway in DN, it was later found that high-glucose-induced TLR4 overexpression could also regulate the expression of Ang II. Pan et al. demonstrated that ACE was regulated by MAPK signaling pathway in renal proximal tubular cells under high-glucose stimulation, along with the increase of renal Ang II level and the development of DN [89]. Ma et al. found TLR4 activation promoted renal inflammation, podocyte and tubular epithelial cell injury, and interstitial fibrosis, and TLR4^−/−^ mice were protected against the development of DN [73]. In addition, Wang et al. revealed that hyperglycemia-activated MD2/TLR4 mediated local RAS activation and Ang II production via an MAPK-dependent manner, which strongly supported that blockage of MD2/TLR4 could significantly attenuated DN via reducing renal RAS activation [90].

**Figure 4:**
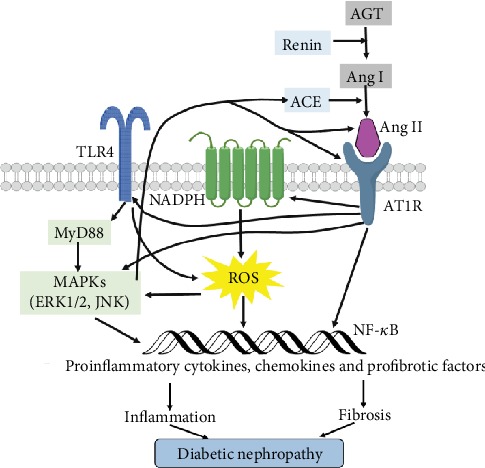
The possible cross-regulation of RAS and TLR4 in mediating the inflammation of DN. By binding to AT1R, Ang II upregulates TLR4 with consequent activation of MyD88/MAPK pathway, resulting in NF-*κ*B activation as well as production of proinflammatory cytokines, chemokines, and profibrotic factors that contribute to diabetic nephropathy. In addition, activated TLR4/MyD88/MAPK signaling cascade enables to induce ACE and AT1 expression, Ang II production, and inflammation in DN.

In this part, we present several reports that support RAS dysregulation affects the innate immune-induced DN. In addition, increased generation of ROS and NADPH oxidase in response to Ang II are markedly inhibited in TLR4-deficient mice. In addition, RAS blockade by using ACE inhibitors or Ang II receptor blockers can downregulate TLR4 expression, resulting in decreasing the expression of proinflammatory cytokines and inhibiting the NF-*κ*B activation as well as slowing the development of DN. These findings suggest a possible cross-regulatory mechanism between AT1R and TLR4. Taken together, Ang II exerts its proinflammatory reactions partly by inducing dysregulation of TLR4-dependent signaling pathways in different renal cells of kidney. And, under the situation of hypertension and/or high-glucose stimulation, there is a possible feedback regulation between RAS components and TLR4 signaling pathway in mediating the initiation and progression of DN (Figure 4).

## 2. Discussion

Previous studies have remarkably revealed the correlation among innate immunity, RAS, and DN. However, the contribution of the RAS system in the inflammatory process of DN, particularly by mediating the expression of TLRs, is less studied. Recently, increasing evidences have shown similar association between the abnormally regulated RAS, increased proinflammatory cytokines production, and NF-*κ*B activation along with increased ROS generation as well as upregulated TLR4 contributing to end-organ damage in DN. And these studies have shown a cross talk between Ang II and TLR4 in mediating these effects within the kidney, liver, heart, vasculature, and brain.

Even though the studies mentioned above strongly suggest that Ang II activates TLR4, it is still unknown how this process might occur in DN. Except for the independent regulation of RAS components or TLR4 activation, whether there is a direct effect of RAS components on TLR4 activation is still unclear. A growing body of evidences indicates that under the stimulation of high glucose, activated TLR4 as well as their endogenous ligands are increased. Subsequently, the activation of TLR4 triggered the initiation of down-stream signaling cascades, which cause the secretion of pro-inflammatory cytokines and activation of NF-*κ*B, resulting in the development of DN. Furthermore, people have also strongly demonstrated that high glucose leads to the activation of RAS, increase of oxidative stress, and ROS upregulation, which induce the occurrence of inflammatory process in DN. In addition, further studies demonstrate that both RAS components and TLR4 signaling pathway attend in the regulation of inflammation during kidney injury and renal fibrosis. As we all know, inflammation-mediated kidney injury and renal fibrosis are two major causes for the development of DN. Therefore, the inflammatory process of DN is finally considered to be strongly linked to the RAS activation and TLR4 signaling pathway dysregulation. Additionally, it observed that activated TLR4 down-stream signaling pathway under high glucose contributed to RAS component upregulation and kidney fibrosis in DN, which supported that the pathogenesis of DN might be caused by the correlation of Ang II dysregulation and TLR4 abnormal expression. By analyzing the previous studies, we hypothesize that there is a feedback regulation between RAS components and TLR4 signaling pathway in mediating the initiation and progression of DN. However, RAS components or TLR4, in which one is the major conductor, and the precise functional mechanisms between them still need to be further investigated. In this review, enough evidences are still deficient to define whether there is a direct effect of Ang II on TLR4 or a feedback regulation in mediating the DN progression. Depending on the studies discussed here, we propose the effects of Ang II on inducing TLR4 activation, via AT1R, producing inflammatory cytokines and oxidative stress during DN. Therefore, understanding the cross-regulatory mechanism between RAS and TLR4 is valuable in the development of new therapeutic strategy for DN.
